# Same-day discharge after appendectomy for acute appendicitis: a systematic review and meta-analysis

**DOI:** 10.1007/s00384-021-03872-3

**Published:** 2021-02-11

**Authors:** Elisabeth M. L. de Wijkerslooth, Jay M. Bakas, Joost van Rosmalen, Anne Loes van den Boom, Bas P. L. Wijnhoven

**Affiliations:** 1 Department of Surgery, Erasmus MC–University Medical Centre, PO Box 2040, 3000 CA Rotterdam, the Netherlands; 2 Department of Biostatics, Erasmus MC–University Medical Centre, Rotterdam, the Netherlands

**Keywords:** Appendicitis, Appendectomy, Same-day discharge, Length of stay, Readmission

## Abstract

**Purpose:**

Patients presenting with acute appendicitis are usually hospitalized for a few days for appendectomy and postoperative recovery. Shortening length of stay may reduce costs and improve patient satisfaction. The purpose of this study was to assess the safety of same-day discharge after appendectomy for acute appendicitis.

**Methods:**

A systematic review was performed according to PRISMA guidelines. A literature search of EMBASE, Ovid MEDLINE, Web of Science, Cochrane Central, and Google Scholar was conducted from inception to April 14, 2020. Two reviewers independently screened the literature and selected studies that addressed discharge on the same calendar day as the appendectomy. Risk of bias was assessed with the ROBINS-I tool. Main outcomes were hospital readmission, complications, and unplanned hospital visits in the postoperative course. A random effects model was used to pool risk ratios for the main outcomes.

**Results:**

Of the 1912 articles screened, 17 comparative studies and 8 non-comparative studies met the inclusion criteria. Most only included laparoscopic procedure for uncomplicated appendicitis. Most studies were considered at moderate or serious risk of bias. In meta-analysis, same-day discharge (vs. overnight hospitalization) was not associated with increased rates of readmission, complication, and unplanned hospital visits. Non-comparative studies demonstrated low rates of readmission, complications, and unplanned hospital visits after same-day discharge.

**Conclusion:**

This study suggests that same-day discharge after laparoscopic appendectomy for uncomplicated appendicitis is safe without an increased risk of readmission, complications, or unplanned hospital visits. Hence, same-day discharge may be further encouraged in selected patients.

**Trial registration:**

PROSPERO registration no. CRD42018115948

**Supplementary Information:**

The online version contains supplementary material available at 10.1007/s00384-021-03872-3.

## Introduction

Acute appendicitis is one of the most frequent surgical emergencies worldwide and is associated with a substantial clinical and financial burden. Appendectomy is mostly performed through laparoscopy, enabling quick recovery of the patient. Reducing length of stay (LOS) may relieve pressure on hospital bed capacity, reduce healthcare costs, and improve treatment satisfaction [1–5]. Many studies have evaluated the safety and feasibility of expedited discharge after appendectomy. However, the terminology and definitions used for early discharge vary greatly [1–11]. Usually, outpatient appendectomy is defined as discharge after appendectomy without hospital admission and ambulatory appendectomy as postoperative LOS of 12 h at most (with or without overnight hospitalization) [1, 3]. Day-case and same-day suggest discharge on the day of surgery, but are often defined as a maximum postoperative LOS of 24 h [2, 12]. Criteria for patient selection and discharge vary as well. Most often only patients with laparoscopic procedure for simple appendicitis (without perforation or necrosis) are considered eligible for same-day discharge. Some studies also selected for patients without concerns of comorbidities or social/organizational contraindications. A recent review of five studies on ambulatory laparoscopic appendectomy among adults demonstrated its feasibility but the authors were concerned about the methodological quality of the included studies [13]. Several other studies have shown the feasibility of same-day discharge (SDD), defined as discharge on the same *calendar* day as appendectomy [5, 8, 9, 14, 15]. Nevertheless, consensus on the safety of same-day discharge after appendectomy has yet to be established [16, 17], and most patients are still hospitalized for 1 or 2 nights after appendectomy for simple appendicitis [5, 18–20]. The aim of this study was to assess the safety of same-day discharge after appendectomy for acute appendicitis by performing a systematic review and critical appraisal of the available literature.

## Methods

### Protocol

A study protocol was established and entered in the International Prospective Register of Systematic Reviews PROSPERO network (registration no. CRD42018115948) [21]. This systematic review was conducted according to the PRISMA guidelines [22]. In addition, the Cochrane Handbook for Systematic Reviews of Interventions [23] and the AMSTAR 2 Checklist were used [24].

### Search strategy

A comprehensive search was performed in EMBASE, Ovid MEDLINE, Web of science, Cochrane Central, Google Scholar, and ClinicalTrials.gov from inception to April 14, 2020. The initial query was developed in consultation with a library scientist. Among other, search terms included “appendicitis,”, “appendectomy,” “hospital discharge,” “ambulatory,” “outpatient,” and “day case.” The complete search strategy is outlined in Online Resource “Appendix A.” The search was limited to articles published in the English language. Manual reference checks were performed in relevant articles.

### Study selection

**Fig. 1:**
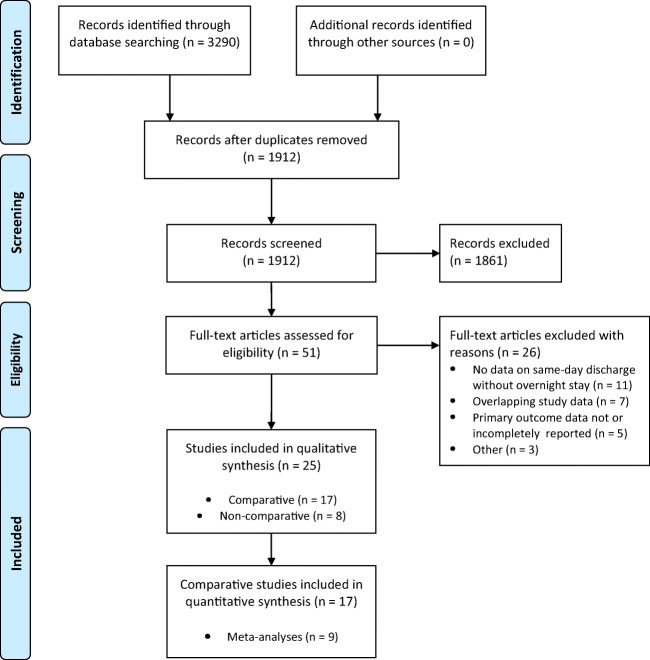
PRISMA flow diagram. From Moher D, Liberati A, Tetzlaff J, Altman DG, The PRISMA Group (2009). Preferred reporting items for systematic reviews and meta-Analyses: the PRISMA statement. PLoS Med 6(7): e1000097. doi:10.1371/journal.pmed1000097

Studies presenting outcome data for patients with same calendar day discharge (SDD) after appendectomy were eligible. In this study, SDD included ambulatory appendectomy, day-case appendectomy, and any other protocol of discharge on the day of appendectomy without overnight hospital stay [1–3]. The following study types were included: randomized controlled trial, prospective observational (cohort) study, retrospective observational (cohort) study, case-control study, and case series. Studies were included if at least one of the main outcomes was reported. Titles and abstracts were first screened for eligibility. Articles were excluded if the abstract revealed no relevance to the subject or if they concerned one of the following: conservative/nonoperative treatment of appendicitis, case reports, and editorials without evaluation data. Two reviewers (EW and JB) independently assessed all non-duplicate articles for inclusion. Disagreements were resolved via negotiated consensus. Subsequently, full-text articles of potentially eligible studies were reviewed, and a final selection of studies was agreed on. If full-text was unavailable, the corresponding author was contacted to request access. Reasons for exclusion after full-text screening are reported in the flowchart (Fig. 1).

### Risk of bias assessment

Two reviewers independently assessed the risk of bias in each comparative study, using the Risk Of Bias In Non-randomized Studies-of Interventions (ROBINS-I) tool [25]. The ROBINS-I tool evaluates the risk of bias in 7 domains: bias due to confounding, bias in selection of participants into the study, bias in classification of interventions, bias due to deviations from intended interventions, bias due to missing data, bias in measurement of outcomes, and bias in selection of the reported results.

### Outcomes

The main outcomes were hospital readmission, complications and unplanned hospital visits within 30 days after appendectomy. Complications were defined as any complication overall or any surgical site complication. Unplanned hospital visits were defined as visits to the Emergency Room (ER) and/or to the outpatient clinic (excluding planned postoperative follow-up appointments).

Secondary outcomes were (radiological or surgical) reinterventions, length of hospital stay, costs, and treatment satisfaction.

### Data extraction and statistical analysis

Outcome data were extracted as well as data on study period and origin, study design, patient selection, number of patients, characteristics of study group, and follow-up time. Data were collected by one reviewer and verified by another. Outcomes are either displayed as reported originally or calculated from the raw reported data. Uncomplicated acute appendicitis was defined as acute appendicitis without findings of necrosis/gangrene or perforation, unless otherwise specified.

Only comparative studies were considered for meta-analysis. Assessment of the study characteristics identified three methodological categories. Some studies compared SDD in a prospective cohort with a historical cohort. Three studies compared SDD to discharge on postoperative day (POD) 1 or 2 and excluded patients discharged after 2 days. This was done to exclude patients with prolonged hospital stay due to immediate complications and/or medical reasons. The third category comprises of studies that compared patients with SDD to patients with overnight stay (for one or more nights) during the same study period. This group of studies was felt to be conceptually different from the other studies, since the control groups included patients that stayed overnight for various reasons that may have affected their chance of adverse outcomes: medical reasons (i.e., nausea, pain, comorbidities, complex type appendicitis), social and organizational reasons (i.e., late surgery, home > 1 h from hospital, no accompanying adult). It was decided to exclude these studies from meta-analysis. The other study categories were considered appropriate for meta-analysis but inappropriate for pooling together due to heterogeneity in study design. Hence, meta-analyses were conducted separately for studies comparing patients in a SDD protocol to historical controls and studies comparing SDD to discharge on POD1-2.

Meta-analyses were performed for the risk ratio (RR) of three outcomes (readmission, complications, and unplanned hospital visits), using a random-effects meta-analysis model. In this model, the Sidik-Jonkman method was used to estimate the between-study variance [26, 27]. The *I*^2^-statistic and Cochran’s *Q* test were used to assess statistical heterogeneity between studies. Meta-analysis was also applied to presented results with adjustment for covariates, based on the published adjusted odds ratios (OR) and confidence intervals in two studies (Cairo et al. adjusted for: age, ASA-class, sex, race, and ethnicity [5]; Grigorian et al. adjusted for age, wound classification, ASA-class, several comorbidities, and steroid use [15]). Results are presented in forest plots. Analyses were performed in R version 3.5.2 [28].

## Results

### Study selection

Literature search identified 1912 non-duplicate articles. After abstract and full-text review, 25 studies, 17 comparative, and 8 non-comparative observational studies were included. The flowchart of the study selection is presented in Fig. 1. The rate of same-day discharge among the cohorts ranged from 22 to 96%. Ten studies included pediatric patients only and ten studies adults only. Five studies included patients from all ages.

### Comparative studies

Characteristics of all comparative studies are shown in Table 1, grouped into three categories according to study design. Five studies compared patients in a prospective SDD protocol to patients from a historical control cohort (with a lower percentage of SDD) [12, 29–32]. Three multicenter retrospective studies compared SDD to discharge on POD 1 or 2 at the latest [5, 15, 33]. The remaining nine studies compared successful SDD to overnight hospitalization for one or more nights [7, 9, 34–40]. Overnight hospitalization occurred for varying reasons of medical, social, and organizational nature. Since these factors may well have affected the outcomes of interest, the latter group of studies was excluded from meta-analysis. Variations in cohort selection criteria, discharge criteria, and reasons for failing SDD are further illustrated in supplementary table S1 (Online Resource ‘Appendix B’).

**Table 1:** Characteristics of comparative studies

Study	Country Study period	Study design	Patient selection					SDD group, *n* (%)	Control group, *n* (%)	Outcomes
			LA/OA	Appendicitis	Age, yrs	Exclusions	*N*			
Studies comparing patients in a SDD protocol to historical controls										
Cash et al. 2012 [29]	U.S.A 2009–2011	Prospective cohort	LA	UAA	≥ 18	Pregnancy	235	116 (49) 85% SDD-PACU	119 (51)^a^ 35% SDD	Readmission Complications
Dubois et al. 2010 [30]	Canada 2005–2007	Retrospective cohort	LA	UAA + CAA	All ages	-	317	161 (51) 45% SDD-PACU pLOS 13.1 h (4.8; 42.3)	156 (49)^a^ % SDD nr pLOS 29.7 h (13.9; 47.5)	Complications Unplanned visits Costs
Lefrancois et al*.* 2015 [31]	France 2013	Prospective cohort	LA	UAA + CAA	All ages	-	652	184 (28) 20% SDD pLOS 41.8 h ± 59.0	468 (72)^a^ 0 % SDD pLOS 47.1 h ± 55.4	Readmission Complications
Putnam et al. 2014 [12]	U.S.A. 2009–2013	Prospective cohort	LA + OA 93% LA	UAA	< 18	-	794	478 (60) 32% SDD pLOS 42 h (17; 31)	316 (40) 7.6% SDD pLOS median 15-18 h	Readmission Complications Unplanned visits Costs
Rosen et al. 2017 [32]	U.S.A. 2014–2016	Prospective cohort	LA	UAA	> 18	Pregnancy, penitentiary ward patients	351	173 (49) 65% SDD-PACU pLOS 9.3 h ± 12.9	178 (51)^a^ % SDD nr pLOS 19.3 h ± 13.2	Readmission Complications Unplanned visitsPatient satisfaction
Studies comparing SDD to discharge on postoperative day 1 or 2										
Cairo et al. 2017 [5]	U.S.A. 2012–2015	Retrospective cohort^M^	LA + OA 95% LA	UAA	< 18	-	20,981	4662 (22)	16,319 (78) Max. 2 nights	Readmission Complications
Grigorian et al. 2019 [15]	U.S.A. 2016–2017	Retrospective cohort^M^	LA	UAA^b^	≥ 18	-	16,931	3988 (24)	12,943 (76) Max. 2 nights	Readmission Complications
Scott et al. 2017 [33]	U.S.A. 2010–2014	Retrospective cohort^M^	LA	UAA^b^	> 18	-	12,703	6710 (53) SDD-PACU	5993 (47) Max. 48 h	Readmissions Wound complications Unplanned visits Costs
Studies comparing SDD to overnight stay for one or more nights										
Aguayo et al. 2014 [34]	U.S.A. 2012–2013	Retrospective cohort	LA	UAA^b^	Children^c^	-	588	128 (22) pLOS 7.3 h ± 2.5	460 (78)^a^ pLOS 22 h ± 11.3	Readmission Complications Unplanned visits
Alkhoury et al. 2012 [35]	U.S.A. 2010-2011	Prospective cohort	LA	UAA + interval	Children^c^	-	158	162 (78) SDD-PACU pLOS 5 h ± nr	45 (22)^a^ pLOS 16 h ± nr	Readmission Complications Unplanned visits Parent satisfaction
Benedict et al. 2018 [9]	U.S.A. 2015–2017	Retrospective cohort	LA	UAA^b^	< 18	-	569	495 (87) pLOS 4 h (3;5)	74 (13)^a^ pLOS 19 h (15;25)	Readmissions Complications
Farach et al. 2014 [36]	U.S.A. 2014	Prospective cohort	LA + OA 76% LA	UAA + CAA	< 21	Pre-existing complex medical conditions, late surgery, inadvertent admission to inpatient unit, social indications	349	185 (53) pLOS 3.1 h ± 1.4	164 (47)_a_ pLOS 66.1 ± 84.8	Complications Costs
Gignoux et al. 2018 [37]	Switzerland 2015–2016	Retrospective cohort	LA	UAA + CAA	All ages	-	185	109 (59) tLOS 8.5 h (3.3;20.5)	76 (41)^a^ tLOS nr	Readmission Complications Unplanned visits
Gurien et al. 2017 [38]	U.S.A. 2015	Retrospective cohort	LA	UAA	≤ 18	-	171	63 (37) SDD-PACU tLOS 3.1 h ± nr	108 (63)^a^ ≥ 1 nights tLOS 14.6 h ± nr	Readmission Complications Unplanned visits Costs
Halter et al. 2016 [7]	U.S.A. 2012-2015	Retrospective cohort	LA	UAA	1–18	Pre-existing medical requirement for admission	236	121 (51) SDD-PACU tLOS 11.8 h ± 2.7	115 (49) Max. 1 night tLOS 24.8 h ± 21.2	Readmission Complications Unplanned visits Costs Family satisfaction
Hussain et al. 2014 [39]	India nr	Prospective cohort	LA	UAA	14–60	Multiple comorbidity, coagulation disorders, adverse anesthetic history, malignancy, ASA-III (uncontrolled) or IV, BMI > 35	30	26 (87) pLOS 9.6 h ± nr	4 (13)^a^ Max. 1 night pLOS 22.0 h ± nr	Readmission Complications Unplanned visits Patient satisfaction
Yu et al. 2017 [40]	U.S.A. 2016-2017	Prospective cohort	LA	UAA	5–18	Pre-existing medical or social requirement for admission	602	185 (31) pLOS 4.4 h (3.1;6.2)	417 (69)^a^ pLOS 17.4 h (14.3;21.8)	Readmission Complications Unplanned visits Costs

*SDD*, same-day discharge; *LA*, laparoscopic appendectomy; *OA*, open appendectomy; *UAA*, uncomplicated acute appendicitis; *CAA*, complicated acute appendicitis; *SDD*-*PACU*, discharge directly from the postanesthesia care unit (recovery room); *ASA*, American Society of Anesthesiologists; *pLOS*, length of stay from operation to discharge (expressed as mean ± sd or median (interquartile range)); *tLOS*, total length of stay from admission to discharge; *nr*, not reported

^a^Reasons for overnight stay summarized in supplementary table S1 (Appendix B)

^b^Uncomplicated acute appendicitis included all unperforated appendicitis in this study (gangrenous or necrotic appendicitis not excluded)

^c^No age limit(s) specified in Methods^M^ Multicenter study

### Risk of bias assessment

The ROBINS-I results are highlighted in supplementary table S2 (Online Resource ‘Appendix B’). The overall risk of bias was considered moderate in five studies, serious in ten studies and critical in two studies.

Table 2 outlines the main outcomes for the comparative studies.

**Table 2:** Primary outcomes of comparative studies

Study	Follow-up duration	Readmissions, *n* (%)			Complications, *n* (%)			Unplanned hospital visits, *n* (%)		
		SDD group	Control group	*p*	SDD group	Control group	*p*	SDD group	Control group	*p*
Studies comparing patients in a SDD protocol to historical controls										
Cash et al. 2012 (29)	2 weeks	0	2 (1.7)	-	6 (5.2)	10 (8.4)	ns^a^	nr	nr	-
Dubois et al*.* 2010 (32)	30 days	nr	nr	-	17 (10.6)	21 (13.5)	0.490	22 (13.7)	24 (15.4)	0.66
Lefrancois et al. 2015 (30)	30 days	16 (8.7)	25 (5.3)	ns^a^	35 (19)	58 (12.9)	0.029^a^	nr	nr	-
Putnam et al. 2014 (12)	30 days	17 (3.6)	4 (1.2)	0.049^a^	13 (2.7)	5 (1.6)	ns^a^	24 (5.0)	6 (1.9)	0.024^a^
Rosen et al. 2017 (31)	2 weeks	3 (1.7)	3 (1.7)	1	6 (3.4)	4 (2.2)	0.54	11 (6.3)	10 (5.6)	0.83
Studies comparing SDD to discharge on postoperative day 1 or 2										
Cairo et al. 2017 (5)	30 days	88 (1.9)	380 (2.3)	0.07	57 (1.2)	261 (1.6)	0.06	nr	nr	-
Grigorian et al. 2019 (15)	30 days	71 (1.8)	297 (2.3)	0.051	41 (10.3)	196 (15.1)	0.022_a_	nr	nr	-
Scott et al. 2017 (33)	30 days	149 (2.2)	183 (3.1)	< 0.005	147 (2.2)^b^	160 (2.7)_b_	ns	847 (12.6)^c^	742 (12.4)^c^	ns
Studies comparing SDD to overnight stay for one or more nights										
Aguayo et al. 2014 (34)	nr	1 (0.8)	6 (1.3)	ns^a^	2 (1.6)	11 (2.4)	ns	6 (4.7)	25 (2.4)	ns
Alkhoury et al. 2012 (35)	2 weeks	4 (2.5)	1 (2.2)	ns	13 (8.0)	3 (6.6)	ns	12 (7.4)	2 (4.4)	ns
Benedict et al. 2018 (9)	nr	8 (2)	3 (4)	0.16	nr^d^	nr^d^	-	nr	nr	-
Farach et al. 2014 (39)	2 weeks	nr	nr	-	5 (2.7)	18 (11)	0.002	nr	nr	-
Gignoux et al. 2018 (40)	30 days	5 (4.6)	7 (9.2)	0.24	13 (11.9)	19 (25)	0.03	13 (11.9)	17 (22.4)	0.07
Gurien et al. 2017 (36)	nr	0	1 (0.9)	-	1 (1.6)	0	-	5 (7.9)	8 (7.4)	0.98
Halter et al. 2016 (7)	30 days	1 (0.8)	3 (2.6)	0.68	1 (0.8)	3 (2.6)	0.35	8 (6.7)	3 (2.6)	0.17
Hussain et al. 2014 (37)	10 days	0	0	-	0	0	-	0	0	-
Yu et al. 2017 (38)	30 days	1 (0.5)	10 (2.4)	0.19	3 (1.6)	13 (3.1)	0.29	8 (4.3)	25 (6)	0.41

*SDD*, same day discharge; *ns*, not statistically significant; *nr*, not reported

^a^Proportions tested for significance (simple *X*^2^ test) based on extracted raw data reported, with α level of 0.05

^b^Wound-related complication instead of any postoperative complication

^c^No statistically significant difference in unplanned ER visits. Rate of postoperative clinic visits (planned + unplanned) did differ: 5460 (81.4%) vs 5121 (85.5%) in the SDD vs. control group (*p* < 0.0001)

^d^Complications reported in the manuscript text, but incomplete data

### Hospital readmission

**Fig. 2:**
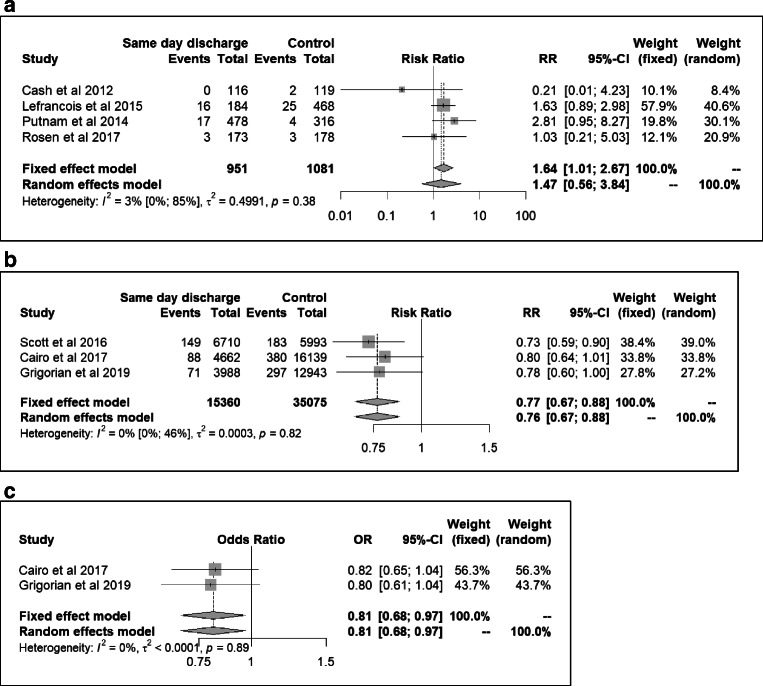
Meta-analyses on the association between SDD and rate of readmission

Fifteen studies with varying duration of follow-up reported readmission rates (Table 2). Readmission after SDD ranged from 0 to 4.6%. One study reported a significantly higher readmission rate for the SDD protocol cohort [12]. Meta-analysis with pooled data from four studies comparing readmission rates for SDD protocol patients vs. historical controls demonstrated a RR of 1.47, 95% CI 0.56 to 3.84 (Fig. 2a). Meta-analysis with pooled data from 3 studies comparing readmission rates for SDD vs. discharge on POD1-2 demonstrated a RR of 0.76, 95% CI 0.67 to 0.88 (Fig. 2b). Meta-analysis with pooled adjusted data from two of the latter studies showed a similar association: OR 0.81, 95% CI 0.68 to 0.97 (Fig. 2c). No statistically significant between-study heterogeneity or between-study variance was observed in any of the meta-analyses (*I*^2^ and Cochran’s *Q* results shown in Fig. 2).

### Postoperative complications

**Fig. 3:**
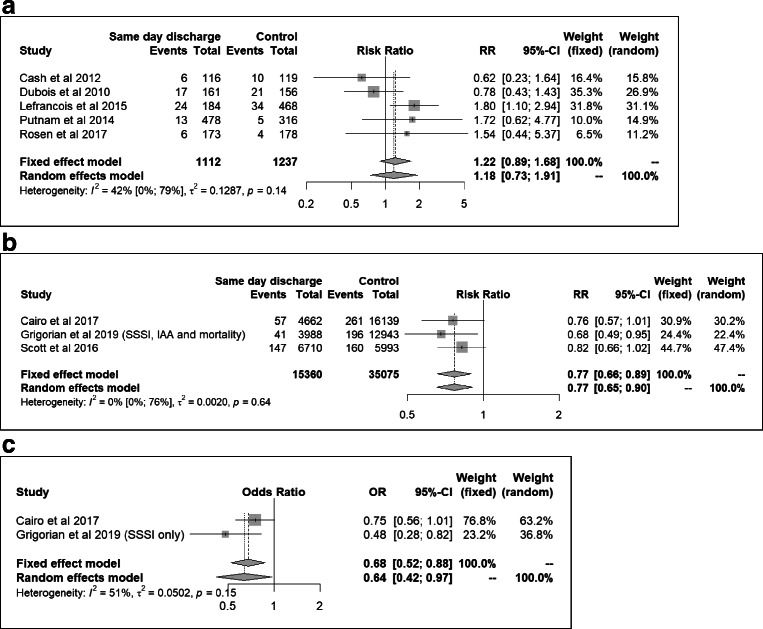
Meta-analyses on the association between SDD and rate of complications

All 17 studies reported postoperative complications. Rates varied between % and 19% (Table 2). There was inconsistency in the definitions used for complications (table S1, Appendix B). One study reported a significantly higher rate of complications for SDD protocol patients [31]. Meta-analysis with pooled data from five studies comparing complication rates for SDD protocol patients vs. historical controls demonstrated a RR of 1.18, 95% CI 0.73 to 1.91 (Fig. 3a). Meta-analysis with pooled data from 3 studies comparing complication rates for SDD vs. discharge on POD1-2 demonstrated a RR of 0.77, 95% CI 0.65 to 0.90 (Fig. 3b). Meta-analysis with pooled adjusted data from two of the latter studies showed a significant association as well: OR 0.64, 95% CI 0.42 to 0.97 (Fig. 3c). No statistically significant between-study heterogeneity was observed in any of the meta-analyses (*I*^2^ and Cochran’s *Q* results shown in Fig. 3).

### Unplanned hospital visits

**Fig. 4:**
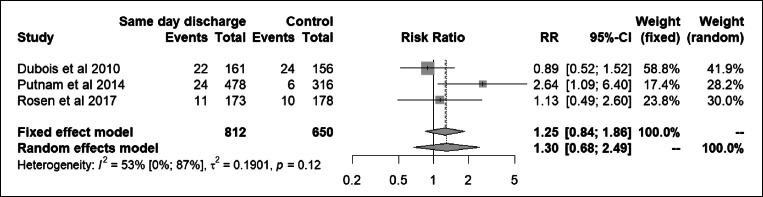
Meta-analysis on the association between SDD and rate of unplanned hospital visits

Eleven studies described unplanned visits to the hospital, ranging from 0 to 12.6% after SDD (Table 2). One study found a significantly higher rate for the SDD protocol group [12]. The remaining studies found no difference in the rate of unplanned visits. Meta-analysis with pooled data from three studies comparing complication rate for SDD protocol patients vs. historical controls showed a RR of 1.30, 95% CI 0.68 to 2.49 (Fig. 4). No statistically significant between-study heterogeneity was observed (*I*^2^ 53%, 95% CI 0–87%, Cochran’s *Q* test *p =* 0.12).

### Other outcomes

Reinterventions—Six comparative studies reported reinterventions to some extent, all showing reoperation occurrence below 1% after SDD [9, 15, 30, 36, 37, 40]. There were no significant differences in reoperation rate between SDD and control group patients (details in Table S3, Appendix B). Another six studies that reported complications, did not present any reintervention in their study cohorts [7, 29, 34, 35, 38, 39].

Length of stay—Thirteen studies reported length of stay, as displayed in Table 1 in hours. Mean postoperative length of stay after SDD ranged from 3.1 ± 1.4 [36] to 9.6 (standard deviation not given) [39] h. Nine studies tested for significance, all reporting a statistically significant reduction in LOS for SDD compared to control groups [7, 9, 12, 30, 32, 35, 36, 39, 40].

Costs—Seven studies performed a cost analysis [7, 12, 30, 33, 36, 38, 40]. Methods of cost analysis were reported in only four studies, and concerned direct hospital-costs, societal costs were outside the scope (details in table S4, Appendix B). All seven studies reported a cost reduction in the SDD group compared to controls, ranging from $323 [30] to $4111 [36]. Three studies showed a statistically significant cost reduction (Table S3, Appendix B).

Treatment satisfaction—Five studies reported treatment satisfaction to some extent [7, 32, 35, 39, 40]. Various short, non-validated surveys were used at different postoperative points in time (details in Table S5, Appendix B). Overall, the studies reported high patient satisfaction after SDD. One study presented satisfaction scores for both SDD protocol patients and historical controls and showed no differences [32].

### Non-comparative studies

Eight non-comparative, observational studies reported outcomes after implementation of an SDD protocol [4, 8, 14, 41–45]. Their characteristics and main results are shown in Table 3. Seven studies reported successful SDD in 80% or more of their selected population. One study reported only 40% SDD [8]. This study only included patients aged 2–18 years. Reported readmission and complication rates ranged from 0 to 6.9% and 0 to 12.8%, respectively. Unplanned hospital visits were observed in 8.1 to 13.2% of patients.

**Table 3:** Characteristics and primary outcomes of non-comparative studies

Study	Country Study period	Study design	Patient selection					SDD *n* (%)	Follow-up duration	Primary outcomes *n* (%)		
			LA/OA	Appendicitis	Age, yrs	Exclusions	*N*			Readmissions	Complications	Unplanned visits
Aubry et al. 2017 (38)	France 2013-2015	Prospective cohort	LA + OA 99% LA	UAA (pre-operatively assessed)	≥ 15	ASA ≥ 3, pregnancy, physical/mental condition preventing participation	102	89 (87)^a^	30 days	2/102 (2)	6/102 (6)	nr
Frazee et al*.* 2016 (42)	U.S.A. 2010-2014	Retrospective cohort	LA	UAA	≥ 18	Pregnancy	563	484 (86)^a^	nr	7/484 (1.5)	38 /563 (7)	nr
Frazee et al. 2017 (43)	U.S.A. nr	Retrospective cohort	LA	UAA	≥ 18	Pregnancy	376	299 (80)^a^	nr	12/376 (3)	18/376 (5)	nr
Gee et al. 2018 (8)	U.S.A. 2016	Prospective cohort	LA	UAA	2–18		961	382 (40)	2 weeks (median)	2/382 (0.5)	49/382 (13)	45/382 (12)
Grelpois et al. 2016 (44)	France 2013-2015	Prospective cohort	LA	UAA	> 18	ASA ≥ 3, pregnancy or breastfeeding, incarceration, legal guardianship	83	76 (92)^a^	30 days	3/76 (4)	13/83 (16)	10/76 (13)
Hobeika et al*.* 2017 (4)	France 2013-2015	Prospective cohort	LA for	UAA + CAA	All ages	St. Antoine score^c^ < 4, no patient consent	102	92 (90)_a_	30 days	7/102 (7)	7/102 (7)	9/102 (9)
Hrad et al. 2015 (45)	U.S.A. 2010-2013	Retrospective cohort	LA	UAA_b_	All ages	Pathological UAA	74	71 (96)^a^	11 days	0	0	6/74 (8)
Sabbagh et al*.* 2019 (14)	France 2016-2017	Retrospective cohort	LA	UAA	All ages	ASA ≥ 3, clinical signs of CAA, living alone or far from a hospital	102	54 (95)_a_	nr	2/54 (3.7)	nr	8/54 (15)

*SDD*, same-day discharge; *LA*, laparoscopic appendectomy; *OA*, open appendectomy; *UAA*, uncomplicated acute appendicitis; *ASA*, American Society of Anesthesiologists; *nr*, not reported; *CAA*, complicated acute appendicitis

^a^Reasons for overnight stay summarized in supplementary table S1 (Appendix B)

^b^Uncomplicated acute appendicitis included all unperforated appendicitis in this study (gangrenous or necrotic appendicitis not excluded)

^c^Score of 1 point per each of the following factors (associated with early discharge in prior retrospective study): BMI < 28 kg/m^2^, WBC < 15.0/μL, C-reactive protein < 30 mg/L, no radiological signs of perforation, and appendix diameter of ≤ 10 mm

With regard to secondary outcomes: reintervention rates ranged from 0 to 3.6% in 7 studies (Table S3, Appendix B), none analyzed costs, and only one study evaluated treatment satisfaction and quality of life (Table S5, Appendix B).

## Discussion

This systematic review demonstrated no increased risk of adverse outcomes following same-day discharge (SDD) after appendectomy. Meta-analyses revealed either no significant association between SDD and rates of readmission, complication and unplanned visits, or a statistically signification association in favor of SDD. Due to substantial clinical and methodological between-study heterogeneity, pooling of data for meta-analysis was limited.

Fifteen of the 17 included comparative studies showed no increase in any adverse outcome after SDD. Two studies reported a statistically significant increase in one or two adverse outcomes after SDD. The differences presented may not be clinically relevant. Hence, same-day discharge seems safe and may be encouraged after careful selection of patients. Results on secondary outcomes (very low rate of reinterventions, significantly reduced postoperative length of stay, indication of reduced costs, no indication of reduced treatment satisfaction), further support SDD. If SDD after appendectomy would be applied more frequently in the future, this will likely reduce hospitalization and associated healthcare costs. With the results of this review in mind, it may be of interest to perform appendectomies early during the day, thereby enabling SDD. Protocols designed to facilitate SDD may be helpful to reduce the need for hospital beds and health care workers, especially during the night.

In contrast to previously published reviews, the present study focused on discharge on the same *calendar* day as the operation and excluded studies that did not explicitly report SDD [15–17]. Sabbagh et al. performed a review on the feasibility of ambulatory surgery (< 12 h length of stay) for several gastrointestinal emergencies in adults [16]. Only three of the 12 included studies on early discharge after appendectomy concerned ambulatory surgery, two of which explicitly reported SDD and are therefore included in the present review. The authors concluded that there is probably a place for ambulatory surgery in clinical practice. Cosse et al. conducted a review on the feasibility of day-case appendectomy for acute appendicitis in adults [2]. They included the same studies as Sabbagh et al. as well as a duplicate publication by Cash et al. [29, 46]. Seven studies reported day-case appendectomy, defined as < 24 h length of stay (hence none were included in the present review). The authors stated that day-case appendectomy was safe and feasible, but more prospective studies should be performed before accepting day-case appendectomy as standard care. Genser et al. also reviewed ambulatory appendectomy and included only three studies, all of which are included in the present review as well. They concluded that ambulatory appendectomy for uncomplicated appendicitis is feasible and may be implemented [17]. Most studies included in these reviews were of retrospective nature. Best evidence would come from prospective trials. A randomized study would be ideal but may not be feasible or ethical. Trejo-Avila et al. recently published a randomized trial related to this topic [10]. In this study, 108 patients were randomized to an enhanced recovery protocol (ERAS) or standard care. Ambulatory management (defined as postoperative LOS < 12 h) was achieved in 90% in the ERAS group vs. 3.4% for standard care [10]. Though this RCT could not be included in the present review as there was no explicit report of (the proportion of) discharge on the same *calendar* day, it does support the findings of the present study. The same authors also performed a systematic review and meta-analysis on ambulatory appendectomy for adult patients [13]. The results are in concordance with ours and represent the best currently available evidence on early discharge after appendectomy. Remarkably, many studies have misleading titles: incorporating the words “same-day discharge,” “outpatient”and/or “ambulatory,” whilst not actually reporting discharge without overnight stay [1]. This was a main reason for excluding full text articles in the present review. Nevertheless, an additional 10 comparative studies were included that were not assessed in the previous reviews, reporting data from both pediatric and adult study populations. Furthermore, eight non-comparative studies were included to summarize evidence on same-day discharge completely. Clinical outcomes after implementation of an SDD protocol in the non-comparative studies were similar to those in the comparative studies.

SDD is feasible and safe in a large proportion of patients. Based on the heterogeneous sample of studies in this review, it is difficult to establish one optimum set of patient selection and discharge criteria for SDD. Selection criteria used in most studies are uncomplicated/unperforated appendicitis and laparoscopic surgery. Twenty-one of the 25 studies in this review excluded open procedures from their cohort. In four studies that included both laparoscopic and open procedures, the proportion of open procedures was low and no separate outcome data were available. Hence, no conclusions can be drawn concerning the safety of SDD after open appendectomy. Both adult and pediatric patients can be considered eligible for SDD after laparoscopic appendectomy. Exclusion of ASA-class III–IV and pregnant patients was often applied as well and seems appropriate. Discharge criteria should entail normal vital signs, ability to tolerate oral intake, ability to ambulate and pain controlled by oral analgesics. Ultimately, the goal will not be to discharge *all* patients on the day of appendectomy, but to improve treatment efficiency by facilitating same-day discharge in a larger proportion of *eligible* patients. A same-day discharge protocol preferably entails a concise set of eligibility criteria that can be assessed preoperatively for the most part. Patients discharged this quickly after surgery should be well informed of relevant signs and symptoms of complications. And adequate (reporting of) follow-up is essential to evaluate the effects of adapting such a protocol.

This study has some limitations. Only non-randomized observational studies were included, which are prone to bias, e.g., due to confounding and selective reporting of results. Meta-analysis was only justified for a limited number of studies. Due to the small number of studies in the meta-analyses, funnel plots for identifying publication bias were not felt to be of added value and statistical between-study heterogeneity (though not observed) cannot be ruled out. Many of the included studies compared SDD patients to a non-matched control group of patients with overnight stay (determined by different medical, social and organizational reasons). Moreover, there was substantial clinical heterogeneity (varying patient selection criteria) as well as methodological heterogeneity (varying study design) among the studies. Lastly, variation in duration of follow-up may have resulted in underreported events. Nonetheless, strengths of the present study are its systematic and extensive nature. A preregistered protocol was adhered to, and the PRISMA guidelines were followed [14], resulting in a large number of recently published studies that was included.

## Conclusion

Current literature provides no indication that same-day discharge is unsafe. Adequate patient selection may be the key to stimulate same-day discharge. It appears safe for most patients undergoing laparoscopic appendectomy for uncomplicated acute appendicitis that meet discharge criteria. Data on costs and treatment satisfaction presented in this review were rather limited. Further implementation of same-day discharge after appendectomy may lower expenses and enhance patient satisfaction.

## Supplementary Information

ESM 1 (PDF 526 kb)

ESM 2 (PDF 889 kb)

## Data Availability

All study data and material are available in the (supplementary) tables and in the original studies.
