# Supplementary material for: Same-day discharge after appendectomy for acute appendicitis: a systematic review and meta-analysis
Source: Int J Colorectal Dis. 2021 Feb 11;36(6):1297–309. doi: 10.1007/s00384-021-03872-3 (PMC8119270; doi:10.1007/s00384-021-03872-3)
Supplement: Supplementary file 1 — (PDF 526 kb) [file 384_2021_3872_MOESM1_ESM.pdf]

APPENDIX I: SEARCH STRATEGY OUTLINE

Search date: April 14, 2020

DATABASE	# of refs	after de-duplication
Embase.com	1569	1544
Medline ovid	772	109
Web of science	619	125
Cochrane CENTRAL	130	54
Google scholar	200	80
**Total**	**3290**	**1912**

Embase.com
('appendectomy'/exp OR 'appendicitis'/de OR (appendectom* OR appendicectom* OR postappendectom* OR postappendicectom* OR appendicit*):ab,ti) AND ('same day discharge'/de OR 'ambulatory surgery'/de OR 'outpatient'/de OR 'hospital discharge'/de OR 'outpatient department'/de OR 'ambulatory care'/de OR (((same-day OR early OR 24h OR 24-h OR 24-hour* OR 12h OR 12-h OR 12-hour* OR within OR timely OR direct*) NEAR/3 discharge*) OR ambulat* OR outpatient* OR out-patient* OR day-surg* OR (day NEXT/1 (case OR care)) OR fast-track OR (short* NEAR/3 stay*)):ab,ti) NOT ([Conference Abstract]/lim) AND [english]/lim

Medline ovid
(Appendectomy/ OR Appendicitis/ OR (appendectom* OR appendicectom* OR postappendectom* OR postappendicectom* OR appendicit*).ab,ti.) AND (Ambulatory Surgical Procedures/ OR Outpatients/ OR Patient Discharge/ OR Ambulatory Care/ OR (((same-day OR early OR 24h OR 24-h OR 24-hour* OR 12h OR 12-h OR 12-hour* OR within OR timely OR direct*) ADJ6 discharge*) OR ambulat* OR outpatient* OR out-patient* OR day-surg* OR (day ADJ (case OR care)) OR fast-track OR (short* ADJ3 stay*)).ab,ti.) AND english.lg.

Web of science
TS=(((appendectom* OR appendicectom* OR postappendectom* OR postappendicectom* OR appendicit*)) AND ((((same-day OR early OR 24h OR "24-h" OR "24-hour*" OR 12h OR "12-h" OR "12-hour*" OR within OR timely OR direct*) NEAR/2 discharge*) OR ambulat* OR outpatient* OR "out-patient*" OR "day-surg*" OR (day NEXT (case OR care)) OR fast-track OR (short* NEAR/2 stay*)))) AND DT=(article) AND LA=(english)

Cochrane CENTRAL
((appendectom* OR appendicectom* OR postappendectom* OR postappendicectom* OR appendicit*):ab,ti) AND ((((same-day OR early OR 24h OR "24-h" OR "24-hour*" OR 12h OR "12-h" OR "12-hour*" OR within OR timely OR direct*) NEAR/3 discharge*) OR ambulat* OR outpatient* OR "out-patient*" OR "day-surg*" OR (day NEXT (case OR care)) OR fast-track OR (short* NEAR/3 stay*)):ab,ti)

Google scholar
appendectomy|appendicectomy|postappendectomy|postappendicectomy|appendicitis
"day|early|24h|hour|12h|timely|direct discharge"|"discharge within"|ambulatory|outpatient|"day-surgery|case|care"|"fast-track"|"short stay"
