# Supplementary material for: Same-day discharge after appendectomy for acute appendicitis: a systematic review and meta-analysis
Source: Int J Colorectal Dis. 2021 Feb 11;36(6):1297–309. doi: 10.1007/s00384-021-03872-3 (PMC8119270; doi:10.1007/s00384-021-03872-3)
Supplement: Supplementary file 2 — (PDF 889 kb) [file 384_2021_3872_MOESM2_ESM.pdf]

SAME-DAY DISCHARGE AFTER APPENDECTOMY FOR ACUTE APPENDICITIS: A SYSTEMATIC REVIEW AND META-ANALYSIS.

APPENDIX B: SUPPLEMENTARY TABLES

E-mail:

Drs. E.M.L. de Wijkerslooth – e.dewijkerslooth@erasmusmc.nl

Contents:

Table S1. Details of included studies page 2

Table S2. ROBINS-I results of comparative studies page 9

Table S3. Reinterventions page 10

Table S4. Costs page 12

Table S5. Treatment satisfaction page 14

References page 16

Table S1. Details of included studies

First author	Patient selection & eligibility for SDD	Type of appendicitis	Discharge criteria	Reasons for overnight stay	Definition complications
Aguayo (1)	>Laparoscopic surgery<br>>Nonperforated AA<br>>Children	Excluded: Perforation defined as a hole in the appendix or fecalith in the abdomen	Not reported	59 (13%) medical<br>18 (3.9%) late arrival<br>2 (0.4%) social<br>381 (83%) clinical care habits	Not specified.
Alkhoury (2)	>Laparoscopic surgery<br>>Acute or interval<br>>UAA<br>>Children	Excluded: perforation or gangrene, or residual abscess or faecolith found at operation	Not reported	5 (11%) medical<br>35 (78%) late operation<br>5 (11%) social	Not specified.
Benedict (3)	>Laparoscopic surgery<br>>Nonperforated AA<br>>Age <18yrs	Excluded: perforation defined as either a hole in the appendix or a fecalith in the abdomen	Not reported	5 (6%) late surgery<br>10 (14%) pain<br>10 (14%) nausa/emesis<br>12 (16%) significant comorbidities<br>37 (50) no specific reason	Not specified.
Cairo (4)	>Laparoscopic or open<br>>UAA<br>>Age <18yrs<br>>Excluding:<br>- Discharge on day ≥ 3	Excluded: Perforated or complicated appendicitis	Not reported	Not reported	Wound complications: eg. superficial surgical site infection (SSI), deep surgical-space infection, deep organ-space infection, and wound disruptions, such as dehiscence)
Cash (5)	>Laparoscopic surgery<br>>UAA<br>>Age ≥18yrs<br>>Excluding:<br>- pregnancy	Excluded: intraoperative findings of perforation, abscess, or gangrenous appendicitis	>Ability to tolerate liquid intake<br>>Ability to ambulate<br>>Pain controlled with oral analgesics<br>>Hemodynamic stability<br>>Adequate respiratory effort<br>>No alteration in mental status from baseline<br>>Ability to urinate<br>>Nausea and vomiting controlled<br>>Physician approval<br>>Appropriate supervision and assistance at home	7 (41%) unrelated medical conditions<br>2 (12%) intraoperative complications<br>8 (47%) physician discretion<br><br>*(all further specified in the paper)*	Not specified.

Abbreviations: CAA, complicated acute appendicitis; UAA, uncomplicated acute appendicitis; NA, not applicable.

Dubois (6)	>Laparoscopic and open<br>>UAA+CAA<br>>All ages<br><br>*Considered for SDD if*:<br>*- Laparoscopic*<br>*- UAA*<br>*- age ≥ 16 and ≤ 65yrs*<br>*- not pregnant, insulin dependent or immunosuppressed*<br>*- adult available to monitor for the first 24h*<br>*- live <30min of a hospital*	Complicated appendicitis was defined as perforation or gangrenous.	>Stable vital signs<br>>Able to tolerate oral intake<br>>Able to void urine<br>>Pain controlled by oral analgesia	27 (17) open/converted surgery<br>6 (4) age <16 or >65<br>2 (1) no responsible adult at home<br>1 (0.6) insulin dependent<br>23 (14) CAA<br>5 (3) postoperative fever<br>2 (1) pain uncontrolled by oral analgesia<br>2 (1) other<br><br>*(numbers above account for the SDD protocol group}*	Any complication within 30 days of the operation, as documented in the patient's health record.
Farach (7)	>Laparoscopic or open<br>>AA or interval<br>>Age <21yrs<br>>Excluded:<br>- pre-existing complex medical conditions not suitable for outpatient surgery<br>- inadvertent admission to the inpatient unit<br>- social indications<br>- late operation	AA was defined as appendiceal hyperemia, dilation, or inflammation without presence of fibrinous exudate, turbid peritoneal fluid, or perforation.<br><br>CAA: intraoperative findings of suppurative, gangrenous or perforated appendicitis.	>Afebrile < 38°C<br>>Hemodynamically stable<br>>Able to tolerate liquids<br>>Adequate pain control<br>>Able to void	46 (28%) excluded preoperatively<br>97 (59%) complex appendicitis<br>9 (5.5%) late operation<br>3 (1.8%) inadequate pain control<br>3 (1.8%) unable to tolerate PO<br>3 (1.8%) fever<br>2 (1.2%) social indications<br>1 (0.6%) medical condition	Not further specified in methods<br><br>Results show 4 categories: superficial wound infection, intra-abdominal abscess, ileus/bowel obstruction and other.
Gignoux (8)	>Laparoscopic surgery<br>>AA<br>>All ages<br>>Excluding:<br>- could not be operated before 5pm or could not be postponed to the next day<br>- home-hospital journey over 1h<br>- patient living alone<br>- severe comorbidities requiring monitoring	All AA.<br><br>Patients with generalized peritonitis or abscess requiring administration of intravenous antibiotics were considered ineligible for SDD.	>No anaphylaxis<br>>Pain that controlled with oral analgesics<br>>No fever<br>>No vomiting	68 (89%) excluded preoperatively<br>3 (4.0%) severe infections<br>1 (1.3%) anaphylaxis<br>2 (2.6%) generalized peritonitis<br>2 (2.6%) excessive pain	Not specified<br><br>Complications were classified according to the modified Clavien system.
Grigorian (9)	>Laparoscopic surgery<br>>Nonperforated AA<br>>Age ≥18yrs<br>>Excluding:<br>- Discharge on day ≥ 3	Excluded: Intraoperative findings of perforation or abscess	Not reported	Not reported	Superficial surgical-site infections (SSSI), post-operative intra-abdominal abscess, mortality

Abbreviations: CAA, complicated acute appendicitis; UAA, uncomplicated acute appendicitis; NA, not applicable.

Gurien (10)	>Laparoscopic surgery<br>>UAA<br>>Age ≤ 18yrs	Excluded: if perforated or gangrenous	Not reported	94 (87%) observation <24h<br>6 (5.6%) surgeon preference<br>4 (3.7%) medical reasons<br>4 (3.7%) social reasons	Not specified
Halter (11)	>Laparoscopic<br>>UAA<br>>Age 1-18yrs<br>>Excluding:<br>- medical indication for postoperative admission	Excluded: evidence of perforation or gangrenous appendicitis	"Patients were deemed safe for discharge home from the recovery room using the same discharge criteria applied to patients discharged following elective same-day outpatient procedures In the Ambulatory Surgery Unit."	Not reported	Not specified
Hussain (12)	>Laparoscopic surgery<br>>UAA<br>>Age 14-60yrs<br>>Excluding:<br>-multiple comorbid diseases<br>-coagulation disorders<br>-adverse anaesthetic history<br>-suspected/proven malignancy<br>-ASA-III or IV<br>- unavailability of competent adult to accompany the patient<br>-BMI >35<br>-Long distance from home (>30min travel)	Uncomplicated symptomatic appendicitis, that is, without abscess, perforation, sepsis and phlegmon formation	a)Stable vital signs >30min<br>b)No new signs/symptoms after the operation<br>c)No active bleeding or oozing<br>d)Minimal nauseau and persistent emesis for <30min<br>e)Orientation in person, time and place<br>f)Pain controllable with oral analgesics<br>g)Passed urine<br>h)No *surgical* complication<br>i)Minimal dizziness after sitting for <10min<br>j)A responsible escort	1 (25%) medical reasons<br>3 (75%) social reasons	Not specified
Lefrancois (13)	>Laparoscopic surgery<br>>UAA+CAA<br>>All ages<br>>Excluded:<br>- interval surgery<br>- stump appendicitis<br><br>*Considered for SDD if: St. Antoine score[*] ≥ 4; UAA; no pregnancy, severe comorbidity or previous pelvic surgery; no severe sepsis or excessive pain; accompanying person; home located <1h transport; sufficient understanding.*	All AA.	>No fever<br>>No pain<br>>No nausea/vomiting	81 (44) score >4<br>45 (69) SDD unit unavailable<br>10 (5.4) not proposed<br>7 (3.8) social/medical contraindication<br>3 (1.6) refusal<br><br>*(numbers above account for the SDD protocol group}*	Any complication within 30 days of operation.

Abbreviations: CAA, complicated acute appendicitis; UAA, uncomplicated acute appendicitis; NA, not applicable.

[*] St. Antoine score was based on BMI, WBC, CRP, no radiological sign of perforation and appendix diameter ≤10mm).

Putnam (14)	>Laparoscopic or open<br>>UAA<br>>Age <18yrs<br>>Excluded:<br>- interval surgery	Excluded: gagrenous or perforated appendicitis at the time of operation.	Not reported.	Not reported.	Postoperative superficial, deep and organ/space SSIs were recorded.
Rosen (15)	>Laparoscopic surgery<br>>UAA<br>>Age ≥ 18yrs<br>>Excluded:<br>- pregnant<br>- wards of the county penitentiary	No evidence of gangrene or perforation.	>Normal vital signs<br>>Adequate pain control<br>>Ability to urinate<br>>Ability to ambulate<br>>Ability to tolerate oral intake<br>>Physician approval<br><br>*(further specified in the paper)*	24 (40) medical reasons<br>23 (38) social reasons<br>13 (22) concern for intraoperative findings<br><br>*(further specified in the paper)*	Not specified
Scott (16)	>Laparoscopic surgery<br>>Nonperforated AA<br>>Age >18yrs<br>>Excluded:<br>- incidental appendectomy<br>- appendectomy concomitant with other procedure<br>- admission >48h	Not specified	Discharge based on a >12 score on the Procedural and Anesthesia Scoring System which is a scoring tool used region-wide to discharge directly from the recovery room after any operation.	Not reported.	Only wound infection reported, no definition specified.
Yu (17)	>Laparoscopic surgery<br>>Uncomplicated<br>>Age 5-18yrs<br>>Excluding:<br>- pre-existing medical conditions requiring inpatient admission<br>- social indications (extensive travel, lack of resources to provide acute care after surgery)<br>- surgery performed between midnight and 7am	Excluded: patients with complex appendicitis (gangrenous or perforated).	Not reported.	32 (39) failed to meet discharge criteria<br>15 (18) surgeon preference for additional IV antibiotics	Any postsurgical condition requiring ED visit, readmission or reoperation. Surgical site infections were defined according to the Centers for Disease Control and Prevention guideline.

Abbreviations: CAA, complicated acute appendicitis; UAA, uncomplicated acute appendicitis; NA, not applicable.

Non-comparative studies					
Aubry (18)	>Laparoscopic or open<br>>UAA<br>>Age ≥ 15yrs<br>>ASA I-II<br>>accompanying adult<br>>place of residence within 1h<br>>Excluding:<br>- physical/mental condition preventing participation<br>- pregnancy<br><br>*Considered for SDD if:*<br>*- no CAA at surgery*<br>*- no complication from anesthesia*	Excluded:presence ofappendiceal or pelvic abscess on radiological examination, radiological pneumoperitoneum and/or general peritoneal effusion.<br><br>CAA defined as abscess, local or general peritonitis discovered during surery.	Not reported.	7 (7) CAA<br>3 (50%) medical reasons<br>3 (50%) social reasons<br><br>*(further specified in the paper)*	Not specified.<br><br>Postoperative complications were graded according to the Clavien-Dindo classification.
Frazee 2016 (19)	>Laparoscopic surgery<br>>UAA<br>>Age ≥ 18yrs<br>>Excluding:<br>- interval surgery<br>- pregnancy	Excluded: gangrenous and ruptured appendicitis	>Ability to tolerate liquid intake<br>>Ability to ambulate<br>>Pain controlled with oral analgesics using VAS<br>>HD stability<br>>adequate respiratory effort<br>>no alteration in mental status from baseline<br>>Ability to urinate<br>>Nausea/vomiting controlled<br>>Physician approval<br>>Appropriate supervision and assistance at home	32 (41) pre-existing conditions<br>10 (13) postoperative morbidity<br>6 (8) physician discretion<br>31 (39) social reasons<br><br>*(further specified in the paper)*	Not specified.
Frazee 2017 (20)	>Laparoscopic surgery<br>>UAA<br>>Age ≥ 18yrs<br>>Excluding:<br>- interval surgery<br>- pregnancy	Excluded: findings of gangrenous or perforated appendicitis	>Ability to tolerate liquid intake<br>>Ability to ambulate<br>>Pain controlled with oral analgesics using VAS<br>>HD stability<br>>adequate respiratory effort<br>>no alteration in mental status from baseline<br>>Ability to urinate<br>>Nausea/vomiting controlled<br>>Physician approval<br>>Appropriate supervision and assistance at home	20 (26) pre-existing conditions<br>21 (27) medical reasons<br>22 (29) social reasons<br>12 (16) physician discretion	Not specified

Abbreviations: CAA, complicated acute appendicitis; UAA, uncomplicated acute appendicitis; NA, not applicable.

Gee (21)	>Laparoscopic surgery<br>>UAA<br>>Age 2-18yrs<br>>Excluding:<br>- interval surgery<br>- additional intra-abdominal pathology (i.e. torsed ovarian cyst)	Excluded: perforated or gangrenous appendicitis	Not reported.	Not reported.	All complications including surgical site infection, abscess, nausea and/or vomiting, intractable pain, readmissions, return to the ER and non-scheduled clinic visits were recorded.
Grelpois (22)	>Laparoscopic surgery<br>>UAA<br>>Age >18yrs<br>>ASA I-II<br>>Excluding:<br>- contraindications for operation or SDD<br>- diagnosis between midnight and 7am or during weekend<br>- pregnancy, breastfeeding<br>- incarceration<br>- living alone or >1h from a hospital<br>- not easy contactable	Excluded: imaging and/or intraoperative findings of abscess, localized or generalized fluid, fecaliths/coproliths	Not reported.	6 (86) medical<br>1 (14) discharge against medical advice	Morbidity (Clavien-Dindo classification)
Hobeika (23)	>Laparoscopic surgery<br>>UAA<br>>All ages<br>>St Antoine score ≥ 4 (based on BMI, WBC, CRP, no radiological signs perforation, diameter ≤ 10mm)<br>>Excluding:<br>- absence of accompanying adult<br>- inavailability day surgery room	Not specified.	Not reported.	4 (4) medical reasons<br>3 (3) patient refusal or end of surgery after 18:00<br>3 (3) no available reason<br><br>*(further specified in the paper)*	Any complication within 30 days of operation.
Hrad (24)	>Laparoscopic surgery<br>>UAA<br>>All ages	Excluded, based on pathology report: normal appendix, chronic, perforated, necrotic/gangrenous appendicitis	>A Modified Aldrete Post Anesthesia Score of 10-12, which does not include the need to void before discharge.<br><br>*(further specified in paper)*	2 (67) medical reasons<br>1 (33) social reasons<br><br>*(further specified in the paper)*	Not reported.

Abbreviations: CAA, complicated acute appendicitis; UAA, uncomplicated acute appendicitis; NA, not applicable.

Sabbagh (25)	>Laparoscopic surgery<br>>UAA<br>>Age >18yrs<br>>ASA I-II<br>>Excluding:<br>- diagnosis between midnight and 7am or during weekend<br>- intraoperative finding of an alternative diagnosis<br>- associated surgical procedure (colectomy, cecectomy, adnexectomy)<br>- insufficient understanding<br>- inavailability of another person to spend the night at the patient's home in the event of an emergency<br>- living > 1h from a hospital<br>- unable to be contacted	Excluding: CAA on the basis of clinical features (sepsis), laboratory signs (renal failure), radiologic or intraoperative signs (abscess, localized or generalized peritonitis, fecaliths, or pneumoperitoneum)	>Ability to tolerate a liquid diet	3 (5) medical reasons	Not reported.

Abbreviations: CAA, complicated acute appendicitis; UAA, uncomplicated acute appendicitis; NA, not applicable.

Table S2. ROBINS-I results of comparative studies

Risk of bias domains

	D1	D2	D3	D4	D5	D6	D7	Overall
Cash *et al.* 2012	x	+	+	+	+	-	-	x
Dubois *et al.* 2010	-	+	+	+	+	-	-	-
Lefrancois *et al.* 2015	x	+	+		+	-	-	x
Putnam *et al.* 2014		+	+		+	-	-	-
Rosen *et al.* 2017	x	+	+	+	+	-	-	x
Cairo *et al.* 2017	-	+	+	+	+	-	-	-
Grigorian *et al.* 2019	-	+	+	+	+	-	-	-
Scott *et al.* 2017	x	+	+	+	+	-	-	x
Aguayo *et al.* 2014	x	+	+	+	+	-	-	x
Alkhoury *et al.* 2012	x	+	+	+	+	-	-	x
Benedict *et al.* 2018	x	+	+	+	+	-	-	x
Farach *et al.* 2014	X	+	+	+	+	-	-	X
Gignoux *et al.* 2018	-	+	+	+	+	-	-	-
Gurien *et al.* 2017	x	+	+	+	+	-	-	x
Halter *et al.* 2015	x	+	+	+	+	-	-	x
Hussain *et al.* 2014	X	+	+	+	+	-	-	X
Yu *et al.* 2017	x	+	+	+	+	-	-	x

Domains
D1 = Bias due to confounding*
D2 = Bias due to selection of participants
D3 = Bias in classification of interventions
D4 = Bias due to deviations from intended interventions†
D5 = Bias due to missing data§
D6 = Bias in measurement of outcomes
D7 = Bias in selection of the reported results[Ω]

Judgement
+ Low
- Moderate
x Serious
X Critical

*Confounding to some extent expected in all studies, few studies performed analysis to adjust for confounders

†In most studies patients were retrospectively included in the intervention or control group based on their date of discharge, which inherently could not be deviated from (patients were either discharged or admitted on the day of surgery).

§In none of the studies loss of follow-up was described. Primary outcomes being readmissions, complications and unplanned hospital visits, it is expected not to be a substantial risk of bias.

[Ω]None of the included studies referred to a prespecified statistical analysis plan. Though no specific indication was found that reported results were selected from larger datasets, this cannot be ruled out.

Table S3. Reinterventions

**First author** *Follow-up*	**Subgroups** (n)	**Reintervention** n (%)	*p* value
Studies comparing patients in a SDD protocol to historical controls			
Cash *2 weeks*	SDD protocol (116)<br>Historical controls (119)	0	-
Dubois *30 days*	SDD protocol (161)<br>Historical controls (156)	1 (0.6) reoperation reported in the SDD protocol group (laparotomy and small bowell resection ue to iatrogenic small bowell injury), no report on possible percutaneous drainage of reported intra-abdominal abscesses.	-
Lefrançois *30 days*	SDD protocol (184)<br>Historical controls (468)	Not reported.	-
Putnam *30 days*	SDD protocol (478)<br>Historical controls (316)	Not reported.	-
Rosen *2 weeks*	SDD protocol (173)<br>Historical controls (178)	Not reported.	-
Studies comparing SDD to discharge on postoperative day 1 or 2			
Cairo *30 days*	SDD (4662)<br>Control group (16139)	Not reported.	-
Grigorian *30 days*	SDD (3988)<br>Control group (12943)	14 (0.3) percutaneous drainages and 0 reoperations<br>37 (0.3) percutaneous drainages and 0 reoperations	ns
Scott *30 days*	SDD (6710)<br>Control group (5993)	Not reported (though in the Methods it is stated that reoperation was examined).	-
Studies comparing SDD to overnight stay for one or more nights			
Aguayo *nr*	SDD (128)<br>Control group (460)	0	-
Alkhoury *2 weeks*	SDD (162)<br>Control group (45)	0	-
Benedict *nr*	SDD (495)<br>Control group (74)	1 (0.2) diagnostic laparoscopy for small bowel obstruction<br>0	-
Farach *2 weeks*	PACU (185)<br>Control group (164)	1 (0.5) exploratory laparotomy with lysis of adhesions for small bowel obstruction, among those completing the protocol	-
Gignoux *30 days*	SDD (109)<br>Control group (76)	1 (0.9) reoperation<br>3 (4.0) reoperations	0.306
Gurien *nr*	PACU discharge (63)<br>Control group (108)	0	-
Halter *30 days*	SDD (121)<br>Control group (115)	0	-
Hussain *10 days*	SDD (26)<br>Control group (4)	0	-
Yu *30 days*	SDD (185)<br>Control group (417)	1 (0.5) reoperation<br>4 (1.0) reoperations	0.69

Non-comparative studies			
Aubry<br>*30 days*	N = 102	1 (1) patient with a pelvic abscess reoperated for drainage.	-
Frazee 2016<br>*nr*	N = 563	0	-
Frazee 2017<br>*nr*	N = 376	1 (0.3) diagnostic laparoscopy to exclude missed enterotomy because of significant postoperative abdominal tenderness	-
Gee<br>*2 weeks*	N = 961	0	-
Grelpois<br>*30 days*	N = 83	3 (3.6) deep abscesses classified as Clavien-Dindo IIIa (2) and IIIb (1)	-
Hobeika<br>*30 days*	N =102	2 (2) patients: 1 transrectal surgical drainage and 1 laparoscopic lavage of pelvic abscesses.	-
Hrad<br>*11 days*	N = 74	0	-
Sabbagh<br>*nr*	N = 57	Unclear from data as presented in paper.	-

SDD, same day discharge; PACU, postanesthesia care unit (recovery room);

Table S4. Costs

First author<br>*Follow-up*	Subgroups (n)	Costs - METHOD	Costs - OUTCOME	*p value*
Studies comparing patients in a SDD protocol to historical controls				
Dubois<br>*30 days*	SDD protocol (161)<br>Historical controls (156)	nr	"After adjusting for inflation to 2007 costs in Canadian dollars, the average cost per patient discharged from the recovery room was $4,845 ± 686; the average cost per patient in the matched control group was $5,168 ± 1,216. This translates to a mean difference of -$323 (95% CI -$670, $23, p = 0.067) per patient."	
Putnam<br>*30 days*	SDD protocol (478)<br>Historical controls (316)	"Cost data were obtained through the hospital cost accounting system. Direct costs were used as the pathway only affected this portion of the total hospital costs, The direct cost of the OR was excluded because the pathway did not change intraoperative practices. The remaining direct costs were averaged for the pre-pathway and pathway cohorts to determine the cost per encounter. Importantly, the costs of readmission and post-discharge events such as SSIs were not obtainable for this study and may limit the applicability of the results."	"the average cost per encounter during the prepathway period was $3,090 ± 996, which decreased to $2719 ± 926 at the time of audit #1 but subsequently increased to $2,988 ± 1,024 at the time of the second audit. Yearly cost savings during the pathway period was $49,053."	
Studies comparing SDD to discharge on postoperative day 1 or 2				
Scott<br>*30 days*	SDD (6710)<br>Control group (5993)	"The cost of treatment is the sum of the hospital's fixed direct cost amount and the hospital variable direct cost amount. Costs for both included both labor and non-labor costs per service cost code allocated in the cost accounting system."	$1,994<br>$2,343	< 0.001
Studies comparing SDD to overnight stay for one or more nights				
Farach<br>*2 weeks*	PACU discharge (185)<br>Control group (164)	nr	"This decrease in inpatient resources resulted in a median reduction in hospital charges of $4111 per patient discharged from the PACU after appendectomy. This resulted in total savings of $760,535 during the 1-year study period."	
Gurien<br>*nr*	PACU discharge (63)<br>Control group (108)	nr	Not reported (reference)<br>+$1007 for admission <24h<br>+$2237 for admission >24h	nr
Halter<br>*30 days*	SDD (121)<br>Control group (115)	"Cost analyses were performed using the hospital cost accounting system and were based on total charges for the entire hospital encounter, including emergency room evaluation, diagnostics, OR and surgical charges, and hospital admission where applicable. No adjustments were made to correct for inflation during the study period."	$10,551 ± 2165<br>$12,691 ± 3507	< 0.001
Yu	SDD (185)		$8073 (6748-9093)	0.002

30 days	Control group (417)	"Costs were compared for the initial admission alone and for an episode of care, which included the initial admission and a period of 30 days after discharge. Direct variable costs, total costs, and payments received (revenue) were analyzed. Margins were estimated by subtracting hospital accounting costs from the payments received. Physician and outpatients pharmacy costs were not included in our economic analysis; nor were education and implementation costs. The institution's internal costing system provided patient-level costs."	$8424 (7207-9725)
Non-comparative studies			
Hobeika<br>*30 days*	N = 102	nr	*"This study did not measure direct costs or charges. Potential cost savings of this approach were estimated using a similar analysis as that used by Frazee. This analysis relied on measurements of the Henry J. Kaiser Family Foundation that reported in 2010 that median cost of 1 day in the hospital was approximately $1910 US. Multiplying this cost by the number of uncomplicated laparoscopic appendectomies performed each year (1910 x 280,000 cases) results in a potential cost savings of $534,800,000/y in the United States."*

SDD, same day discharge; PACU, postanesthesia care unit (recovery room); nr, not reported; ns

Table S5. Treatment satisfaction

**First author** *Follow-up*	**Subgroups** (n)	**Treatment satisfaction - METHOD**	**Treatment satisfaction - OUTCOME**
Studies comparing patients in a SDD protocol to historical controls			
Rosen *2 weeks*	SDD protocol (173)<br>Historical controls (178)	"Satisfaction surveys using a Likert scale (range 1 to 4, with a score of 1 indicating extreme disagreement and 4 denoting extreme agreement) were distributed to all patients at their 2-week follow-up appointment or via phone."	"Responses from both groups showed no statistically significant difference and the median response scores of both groups were identical. The survey covered ability to resume daily activities (median 3 for both groups), pain control (median 4), and understanding of their treatment plan (median 4). Same-day discharge patients were asked if they were nervous about the protocol (median 3), if they were happy with the protocol (median 3), and if they would want a same-day discharge if they could do it over again (median 3). Overall ranking of the hospital (scale 1 to 10) yielded a median score of 9 in both the control and outpatient groups. Response rate for the satisfaction surveys was 55%."
Studies comparing SDD to overnight stay for one or more nights			
Alkhoury *2 weeks*	SDD (162)<br>Control group (45)	"At the postoperative visit 2 to 3 weeks later, parents completed a 1-page survey that asked about their satisfaction with same-day discharge, in addition to information about pain control, return to normal activities, and postoperative problems. The pertinent portion of the survey that relates to parental satisfaction with same-day discharge is given in Table 1."<br>Parent satisfaction survey questions:<br>1.Immediately after surgery, how did you feel about going home on the same day? A) Happy to go home, b) Nervous, but we did fine, c) I wouldn't want to do it again.<br>2. In retrospect, how do you feel now? A) It was the right thing to do, b) It was OK to go home on the same day, but I'm not sure it was best, c) I would not want to do it again.<br>3.Feel free to add other comments regarding your child's surgery."	"Overall, parents were satisfied with their child's expeditious discharge. At the postoperative office visit, 141 of 162 parents (87.0%) said that immediately following the surgery they had been pleased with same-day discharge, whereas 13 parents (8.0%) indicated they felt nervous but were ultimately satisfied; 8 parents (4.9%) were not sure early discharge was best. In retrospect, satisfaction rose to 95.0% at the time of the postoperative office visit, with 154 parents stating that same-day discharge was desirable. Only 1 parent would insist on admission if faced with the situation again."
Halter *30 days*	SDD (121)<br>Control group (115)	"All patients' families were contacted by phone within 2 weeks of surgery to conform there had been an uneventful recovery and to inquire about any issues that might require a follow-up visit in the office. For those patients discharged from the recovery room, families were also mailed a six-question survey regarding their experience."	"With respect to patient satisfaction, surveys were sent to families of all SDD patients, with a total of 32 responses (26% response rate). When families were asked "At the time of discharge, how did you feel about taking your child home the same day following surgery?" the majority (59%) responded "Happy to go home" and an additional 28% responded "Nervous, but OK". Overall, almost 80% replied

			that they would prefer to be discharged from the recovery room in similar circumstances in the future.”
Hussain<br>*10 days*	SDD (26)<br>Control group (4)	nr	“At the time of discharge all patients (100%) were highly satisfied.”
Yu<br>*30 days*	SDD (185)<br>Control group (417)	“All SDD patients were contacted by telephone within 24 hours of discharge to assess their recovery by the surgical team or research staff. A telephone script and a standardized same day discharge appendectomy phone follow-up template were created for documentation within our EMR. Alarming symptoms would prompt the caller to notify the on-call physician and document this within the template.”	“Average satisfaction of recent hospitalization reported was 9.4/10 (range 5-10). 88% reported patient satisfaction of 8 or higher and 76% gave a score of 10/10. There was 100% satisfaction with receiving a telephone follow-up.”
Non-comparative studies			
Grelpois<br>*30 days*	N = 83	“[..] end points [..] were [..] patient satisfaction, and quality of life (according to the 36-Item Short-Form Health Survey administered on discharge).”	“[..] 99% of the patients (n = 82) were satisfied with DCS. The only unsatisfied patient had not wanted to wait for the surgeon before being discharged. The 36-Item Short-Form Health Survey evidenced good quality of life.”

REFERENCES

1. Aguayo P, Alemayehu H, Desai AA, et al. Initial experience with same day discharge after laparoscopic appendectomy for nonperforated appendicitis. J Surg Res. 2014;190(1):93-7.
2. Alkhoury F, Malvezzi L, Knight CG, et al. Routine same-day discharge after acute or interval appendectomy in children: A prospective study. Arch Surg. 2012;147(5):443-6.
3. Benedict LA, Sujka J, Sobrino J, et al. Same-day discharge for nonperforated appendicitis in children: an updated institutional protocol. J Surg Res. 2018;232:346-50.
4. Cairo SB, Raval MV, Browne M, et al. Association of same-day discharge with hospital readmission after appendectomy in pediatric patients. JAMA Surg. 2017;152(12):1106-12.
5. Cash CL, Frazee RC, Abernathy SW, et al. A prospective treatment protocol for outpatient laparoscopic appendectomy for acute appendicitis. J Am Coll Surg. 2012;215(1):101-5.
6. Dubois L, Vogt KN, Davies W, Schlachta CM. Impact of an outpatient appendectomy protocol on clinical outcomes and cost: A case-control study. J Am Coll Surg. 2010;211(6):731-7.
7. Farach SM, Danielson PD, Walford NE, et al. Same-day discharge after appendectomy results in cost savings and improved efficiency. Am Surg. 2014 Aug;80(8):787-91.
8. Gignoux B, Blanchet MC, Lanz T, et al. Should ambulatory appendectomy become the standard treatment for acute appendicitis? World J Emerg Surg. 2018;13:28.
9. Grigorian A, Kuza CM, Schubl SD, et al. Same-Day Discharge after Non-Perforated Laparoscopic Appendectomy Is Safe. J Invest Surg. 2019.
10. Gurien LA, Burford JM, Bonasso PC, Dassinger MS. Resource savings and outcomes associated with outpatient laparoscopic appendectomy for nonperforated appendicitis. J Pediatr Surg. 2017;52(11):1760-3.
11. Halter JM, Mallory B, Neilson IR, Langer M. Same-Day Discharge Following Laparoscopic Appendectomy for Uncomplicated Acute Appendicitis as a Measure of Quality in the Pediatric Population. J Laparoendosc Adv Surg Techn. 2016;26(4):309-13.
12. Hussain A, Singh S, Singh Ahi K, Singh M. Status of Day Care Laparoscopic Appendectomy in Developing Countries. int sch res notices. 2014;2014:502786.
13. Lefrancois M, Lefevre JH, Chafai N, et al. Management of acute appendicitis in ambulatory surgery: Is it possible? How to select patients? Ann Surg. 2015;261(6):1167-72.
14. Putnam LR, Levy SM, Johnson E, et al. Impact of a 24-hour discharge pathway on outcomes of pediatric appendectomy. Surgery. 2014;156(2):455-61.
15. Rosen DR, Inaba K, Oh PJ, et al. Outpatient Laparoscopic Appendectomy: Feasible in a Public County Hospital? J Am Coll Surg. 2017;224(5):862-7.
16. Scott A, Shekherdimian S, Rouch JD, et al. Same-Day Discharge in Laparoscopic Acute Non-Perforated Appendectomy. J Am Coll Surg. 2017;224(1):43-8.
17. Yu YYR, Smith CM, Ceyanes KK, et al. A prospective same day discharge protocol for pediatric appendicitis: Adding value to a common surgical condition. Journal of Pediatric Surgery. 2018 Jan;53(1):36-41.
18. Aubry A, Saget A, Manceau G, et al. Outpatient Appendectomy in an Emergency Outpatient Surgery Unit 24 h a Day: An Intention-to-Treat Analysis of 194 Patients. World J Surg. 2017 Oct;41(10):2471-9.
19. Frazee RC, Abernathy SW, Isbell CL, et al. Outpatient Laparoscopic Appendectomy: Is It Time to End the Discussion? J Am Coll Surg. 2016;222(4):473-7.
20. Frazee R, Burlew CC, Regner J, et al. Outpatient laparoscopic appendectomy can be successfully performed for uncomplicated appendicitis: A Southwestern Surgical Congress multicenter trial. Am J Surg. 2017;214(6):1007-9.
21. Gee K, Ngo S, Burkhalter L, Beres AL. Safety and feasibility of same-day discharge for uncomplicated appendicitis: A prospective cohort study. J Pediatr Surg. 2018;53(5):988-90.
22. Grelpois G, Sabbagh C, Cosse C, et al. Management of Uncomplicated Acute Appendicitis as Day Case Surgery: Feasibility and a Critical Analysis of Exclusion Criteria and Treatment Failure. J Am Coll Surg. 2016;223(5):694-703.
23. Hobeika C, Hor T, Chereau N, et al. Day Surgery for Acute Appendicitis in Adults: A Prospective Series of 102 Patients. Surg Laparosc Endosc Percutan Tech. 2017 Jun;27(3):158-62.
24. Hrad V, Waisbren SJ. Results of Immediate Discharge From Postanesthesia Care Unit to Home After Laparoscopic Appendectomy for Acute Nonperforated Appendicitis. Surg Laparosc Endosc Percutan Tech. 2015 Aug;25(4):343-6.
25. Sabbagh C, Masseline L, Grelpois G, et al. Management of Uncomplicated Acute Appendicitis as Day Case Surgery: Can Outcomes of a Prospective Study Be Reproduced in Real Life? J Am Coll Surg. 2019;229(3):277-85.
